# Ultrasound, a new adjuvant method for treating acute Monteggia fracture in children

**DOI:** 10.1186/s13018-023-04075-y

**Published:** 2023-08-11

**Authors:** YongFei Fan, QiXin Liu, XueDi Yu, JianQiang Zhang, Wei Wang, ChaoYu Liu

**Affiliations:** 1 Department of Orthopaedic Surgery, Fuyang People’s Hospital Affiliated to Anhui Medical University, Anhui Spinal Deformity and Clinical Medical Research Center, Fuyang People’s Hospital, Fuyang, 236000 Anhui People’s Republic of China; 2 Department of Ultrasound, Fuyang People’s Hospital Affiliated to Anhui Medical University, Fuyang People’s Hospital, Fuyang, 236000 Anhui People’s Republic of China

**Keywords:** Monteggia fracture, Ultrasound, Children, Nerve injury

## Abstract

**Purpose:**

This study aims to evaluate the feasibility of using ultrasound-guided Kirschner wire or elastic intramedullary nail for fixation in the treatment of acute Monteggia fracture in children.

**Methods:**

A retrospective analysis was conducted on 31 cases of acute Monteggia fracture in children treated with ultrasound-guided Kirschner wire or elastic intramedullary nail fixation between April 2020 and December 2022, including 14 cases of Kirschner wire fixation and 17 cases of elastic intramedullary nail fixation. During the operation, soft tissue compression and nerve and vascular injuries were explored, fracture reduction was performed under ultrasound guidance, and operation time was recorded. After the operation, X-ray examination was conducted to assess the quality of fracture reduction. At the last follow-up, the flexion, extension, pronation, and supination angles of both affected and unaffected elbow joints were measured, and the Mayo score was used to evaluate elbow joint function.

**Results:**

The average duration of surgery was 50.16 ± 19.21 min (ranging from 20 to 100 min). Based on the evaluation criteria for assessing reduction quality, 28 cases were deemed excellent, while 3 cases were considered good. After immobilization with long-arm cast for 4–6 weeks postoperatively, elbow and forearm rotation exercises were performed. Kirschner wires were removed after an average of 6.64 ± 0.93 weeks (ranging from 6 to 9 weeks) postoperatively, and elastic intramedullary nails were removed after an average of 5.12 ± 1.54 months (ranging from 4 to 10 months) postoperatively. The average follow-up time was 19.13 ± 11.22 months (ranging from 4 to 36 months). During the final follow-up, the affected limb’s range of motion in flexion, extension, pronation, and supination was (141.16 ± 4.24)°, (4.61 ± 2.81)°, (84.52 ± 3.74)°, and (84.23 ± 3.69)°, respectively. There was no notable variance when compared to the healthy limb, which had a range of motion of (141.81 ± 2.99)°, (4.81 ± 2.50)°, (85.61 ± 3.12)°, and (85.03 ± 2.73)° (*P* > 0.05). The Mayo Elbow Performance index classified 29 cases as excellent and 2 cases as good.

**Conclusion:**

Ultrasound-guided Kirschner wire or elastic intramedullary nail fixation can be used for the treatment of acute Monteggia fracture in children, which can explore the surrounding nerves, blood vessels, and soft tissue compression, reduce the difficulty of reduction, and cause minimal trauma. It can greatly reduce the risk of radiation exposure and complications such as vascular and nerve injury during the operation.

## Introduction

Monteggia fracture is a rare injury and typically occurs in children aged 4 to 10, accounting for about 1% of all upper limb fractures in children [1, 2]. If the fracture is not detected or timely treated, it may lead to elbow joint dysfunction, chronic pain, degenerative arthritis, and other complications [3, 4].

Traditional manual reduction and cast immobilization are the first choice for treating acute Monteggia fracture. However, failure of reduction and redislocation still occur frequently [5]. Surgical treatment is a more appropriate option for unstable ulnar oblique fracture, completely displaced transverse fracture, and unstable radial head after reduction. The surgical treatment of ulnar fracture mainly includes Kirschner wire fixation, elastic stable intramedullary nail (ESIN) fixation, plate fixation, and external fixation [5–7]. Among them, Kirschner wire and ESIN fixation are widely used due to their less invasive procedures.

Intraoperative fluoroscopy is the main way to guide and evaluate the quality of reduction, which often requires multiple exposures and significantly increases the patient's radiation exposure. In recent years, with the increasing application of ultrasound technology in pediatric orthopedics, its advantages have gradually emerged. Ultrasound can assist in fracture reduction and at the same time identify soft tissue compression and nerve and vascular injuries [8], which make it easier to formulate subsequent treatment plans. Therefore, the aim of this study was to investigate our results following ultrasound-guided closed reduction and fixation methods in acute Monteggia lesions.

## Patients and methods

This retrospective analysis included data from 31 cases of acute Monteggia fracture treated with ulnar reduction using either the Kirschner wire or elastic intramedullary nail under ultrasound guidance from April 2020 to December 2022. The study was approved by the hospital ethics committee, and the clinical data of the children were used with the consent of their guardians. A total of 31 patients were included in this study, including 18 males and 13 females, with an average age of 6.58 ± 2.73 years (range 5–14 years) and a time from injury to surgery of 2.22 ± 1.73 days (range 1–10 days). There were 16 left-sided and 15 right-sided fractures, of which six were open Gustilo I fractures, five had radial nerve injuries, one had an avulsion fracture of the olecranon process of the ulna, and one had distal fractures of both the ulna and radius. According to the Bado classification, there were 13 cases of type I, five cases of type II, 10 cases of type III, and three cases of type IV. Ulnar fractures were fixed with Kirschner wire in 14 cases and elastic intramedullary nail in 17 cases (Table 1).

**Table 1:** Patients’ preoperative, intraoperative, and postoperative data $(\overline{x} \pm s)$

	Preoperative	Intraoperative	Postoperative
Age (years)	6.58 ± 2.73		
Sex			
Male	18		
Female	13		
Affected side			
Left	16		
Right	15		
Complication			
Ulnar olecranon fracture	1		
Fracture of distal radius and ulna	1		
Radial nerve injury	5		
Bado type			
I	13		
II	5		
III	10		
IV	3		
Time from injury to surgery (min)		2.22 ± 1.73	
Surgery times (min)		50.16 ± 19.21	
Kirschner wire		14	
ESIN		17	
Follow-up time (month)			19.13 ± 11.22
Kirschner wire removal time (week)			6.64 ± 0.93
ESIN removal time (month)			5.12 ± 1.54
Reset evaluation criteria			
Excellent			28
Good			3
Fair			0
Poor			0
MEPI*			
Excellent			29
Good			2
Fair			0
Poor			0

*MEPI, Mayo Elbow Performance Index

### Surgical procedure

**Fig. 1:**
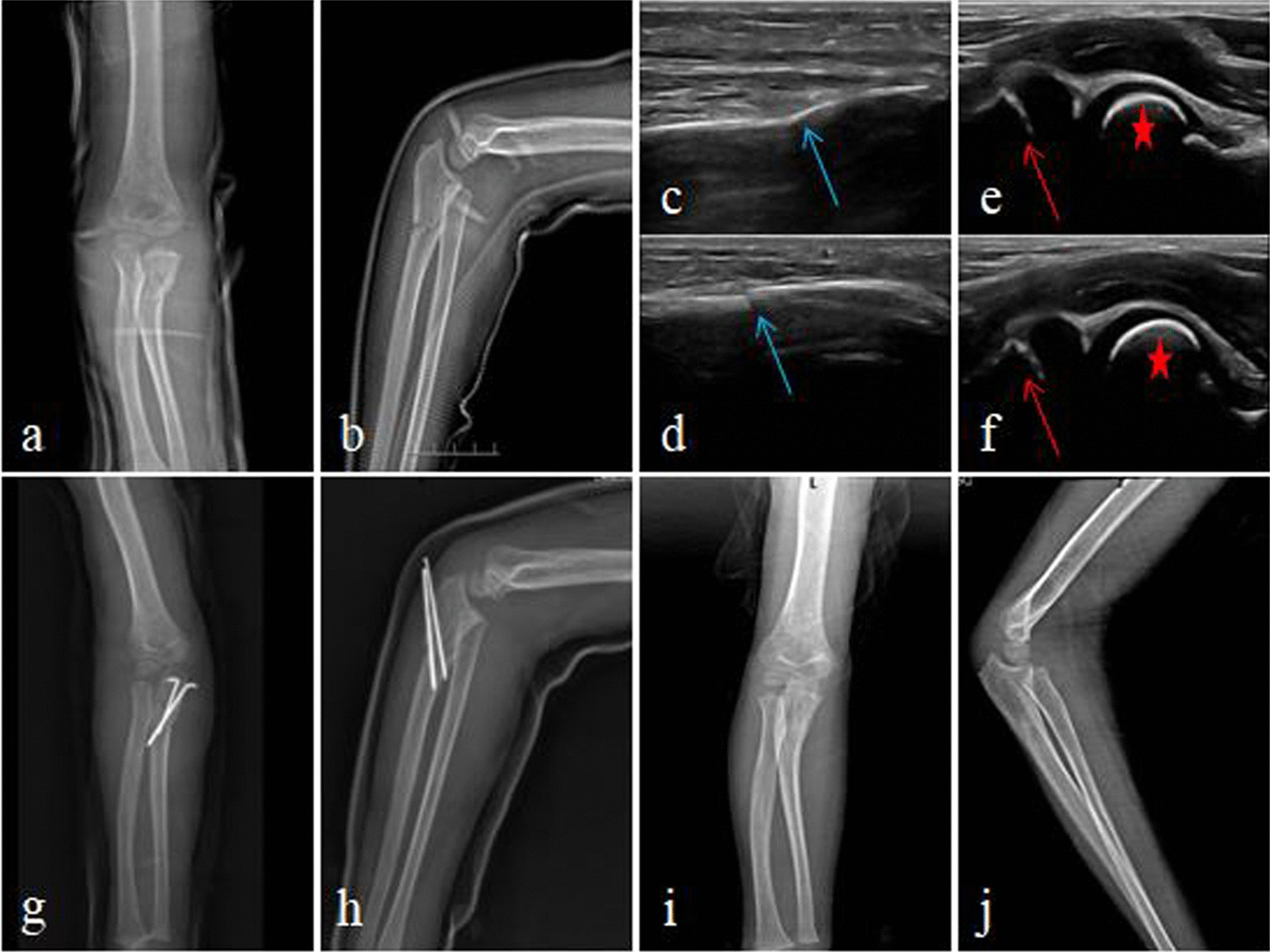
**a**, **b** Preoperative X-ray; **c–f** treatment of acute Monteggia fracture under ultrasonographic guidance (blue arrow—ulna fracture, red arrow—radial head, asterisk—capitulum humeri); **g**, **h** X-ray on the first day after surgery; **i**, **j** X-ray after removal of internal fixation

**Fig. 2:**
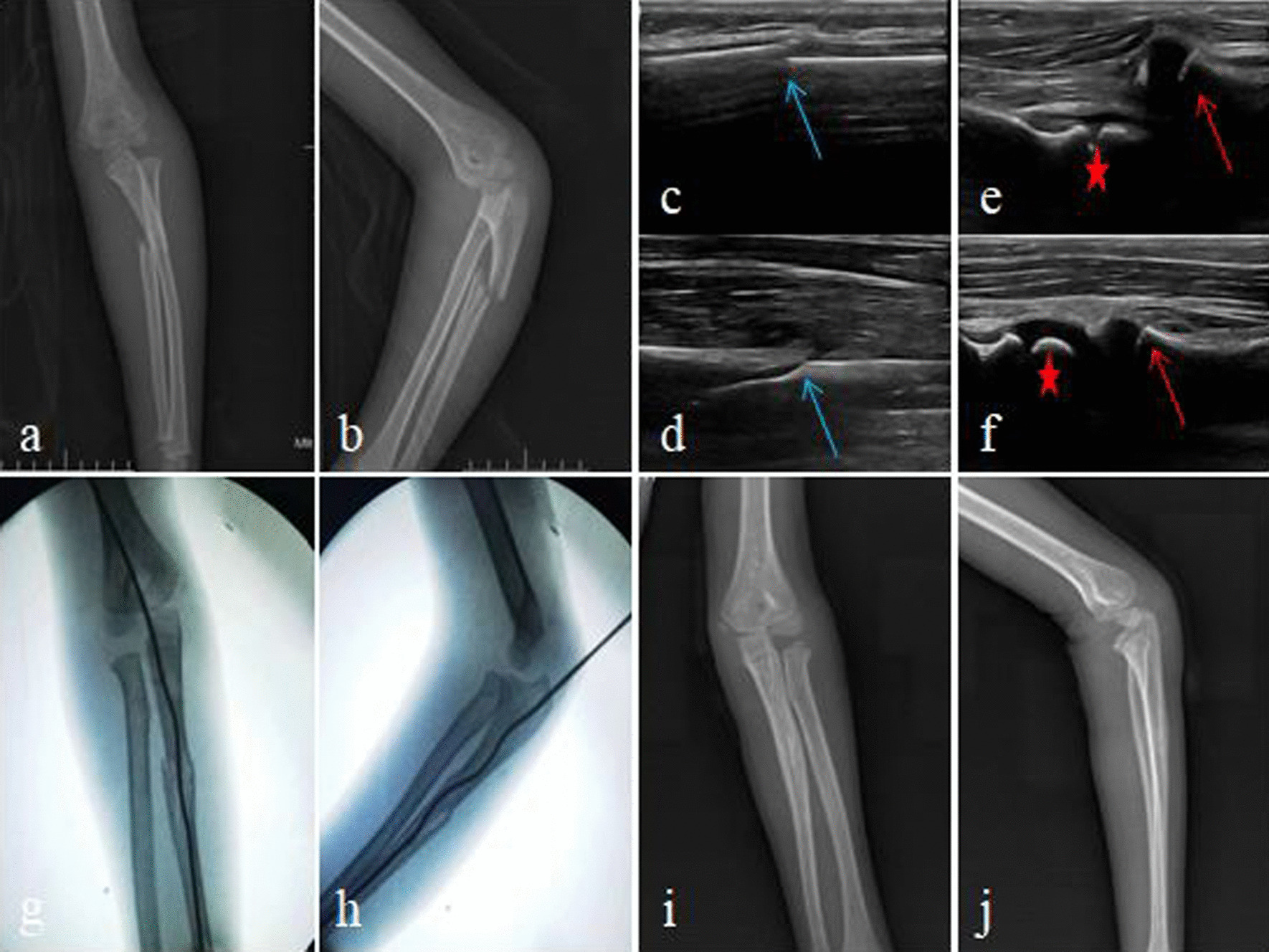
**a**, **b** Preoperative X-ray; **c**–**f** treatment of acute Monteggia fracture under ultrasonographic guidance (blue arrow—ulna fracture, red arrow—radial head, asterisk—capitulum humeri); **g**, **h** X-ray on the first day after surgery; **i**, **j** X-ray after removal of internal fixation

All surgeries were performed under general anesthesia by the same surgical team. Ultrasound gel was applied to the surface of the ultrasound probe, and a sterile disposable plastic film was used to protect the connecting wire and probe. The probe handle was tightly wrapped with a sterile bandage. Sterile saline or medical alcohol was applied to the surface film of the probe for ultrasound examination of the elbow and fracture site. Traction was used to reduce the radial head, and ultrasound was used to detect soft tissue impingement in the humeroradial joint in cases that are difficult to reduce or maintain after reduction. If impingement was detected, a Kirschner wire was used to dislodge the impinging soft tissue. The ulna fracture was reduced under ultrasound guidance, and percutaneous Kirschner wire or elastic intramedullary nail was used for fixation, depending on the type and location of the fracture (Figs. 1, 2). Ultrasound was used again to check the reduction of the humeroradial joint and ulna fracture, as well as the stability of the radial head during elbow extension, flexion, and forearm rotation. Finally, fluoroscopy was used to further verify the reduction and internal fixation. In cases where the elastic intramedullary nail was used, its diameter was approximately 2/3 of the narrowest diameter of the medullary cavity, and it was pre-bent precisely into an S shape, with the two vertices of the S fixed on the proximal and distal segments of the fracture [9] (Fig. 2). No cases of difficulty in reducing the radial head due to impingement of the joint capsule or annular ligament were detected during ultrasound examination. In five cases, uneven degeneration of the radial nerve was observed at the fracture site, but continuity of the nerve was maintained.

### Postoperative management

A long-arm cast was used to immobilize the affected arm for 4–6 weeks. After the cast was removed, elbow flexion and extension as well as forearm rotation exercises were performed to improve joint function. If the fracture healing is unsatisfactory, a short-arm cast was used for an additional 2 weeks while continuing elbow joint exercises. The surgery time was recorded, and the reduction quality was assessed based on X-ray examination during the follow-up. Reduction evaluation criteria were as follows: excellent, no lateral displacement or displacement less than 1/4 and no angulation or angulation less than 5°; good, lateral displacement between 1/4 and 2/3 and angulation between 5° and 10°; and poor, lateral displacement greater than 2/3 and angulation greater than 10° [10]. The occurrence of complications such as redislocation of the radial head, Kirschner wire tail irritation, compartment syndrome, and myositis ossificans as well as nerve recovery status are recorded. The Mayo Elbow Performance index was used to evaluate elbow joint function on the affected side during the last follow-up and compared with the flexion, extension, and rotation angles of the healthy side.

### Statistical analysis

SPSS 26.0 (version 26.0; IBM, Armonk, NY, USA) was used for statistical analysis. Quantitative data were expressed as means ± standard deviations (SD). The comparison between the affected side and the healthy side was performed using a paired sample *t*-test. *P* < 0.05 was considered significant.

## Results

**Fig. 3:**
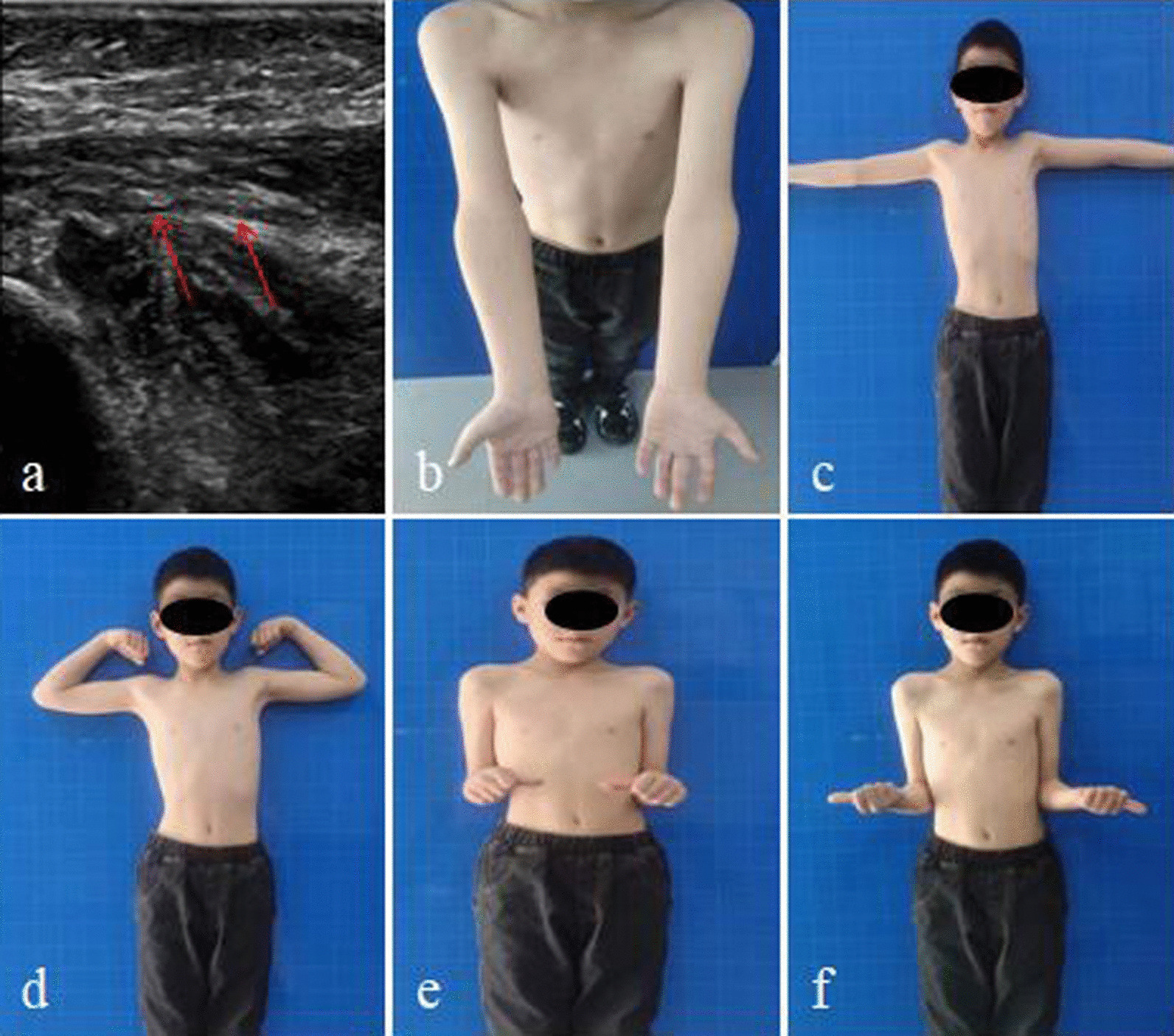
A 7-year-old child with radial nerve injury (red arrow): **a** uneven degeneration of the radial nerve was observed under ultrasonographic guidance; **b–f** the flexion, extension, pronation, and supination functions of the affected elbow joint at the last follow-up after recovery from acute Monteggia fracture in the child

All patients underwent closed reduction and percutaneous Kirschner wire or elastic intramedullary nail under ultrasound guidance, with an average operation time of 50.16 ± 19.21 min (range, 20 to 100 min). X-ray examination was performed one day after the surgery, and there were 28 excellent and 3 good cases based on the reduction assessment standard. The Kirschner wires were removed at an average of 6.64 ± 0.93 weeks (range, 6 to 9 weeks) after the surgery, and the intramedullary nails were removed at an average of 5.12 ± 1.54 months (range, 4 to 10 months) after the surgery. The mean follow-up time was 19.13 ± 11.22 months (range, 4 to 36 months). All patients with radial nerve injury recovered well, and there were no complications such as redislocation of the radial head, needle tail irritation, compartment syndrome, or myositis ossificans. During the last follow-up, 29 cases were rated excellent and 2 cases were rated good based on the Mayo Elbow Performance index for elbow joint function evaluation (Table 1) (Fig. 3). There was no statistically significant difference in the range of elbow flexion, extension, pronation, and supination angles between the affected and healthy sides (*P* > 0.05) (Table 2).

**Table 2:** Comparison of the angles of flexion, extension, pronation, and supination between the affected and contralateral sides at the last follow-up $(\overline{x} \pm s)$

	Flexion (°)	Extension (°)	Pronation (°)	Supination (°)
Contralateral side	141.81 ± 2.99	4.81 ± 2.50	85.61 ± 3.12	85.03 ± 2.73
Affected side	141.16 ± 4.24	4.61 ± 2.81	84.52 ± 3.74	84.23 ± 3.69
*P* (value)	0.449	0.693	0.322	0.385

## Discussion

Monteggia fracture is often caused by indirect violence, involving a fracture of the upper 1/3 of the ulnar bone combined with dislocation of the radial head, which is particularly common in children [11]. Based on the severity of the ulnar fracture and the extent of dislocation of the radial head, Bado classified Monteggia fracture into four types [12]: Type I involves an angular fracture of the ulna shaft with anterior dislocation of the radial head; Type II involves an angular fracture of the ulna shaft with posterior or posterior–lateral dislocation of the radial head; Type III involves an ulnar metaphyseal fracture with lateral or anterior–lateral dislocation of the radial head; and Type IV involves a fracture of both the ulna and radius with anterior dislocation of the radial head. Among children with Monteggia fracture, Type I injury is the most common, accounting for approximately 70%, followed by Type III injury, while Type II and IV injuries are less common [13]. Radial nerve injury is a common concomitant injury in Monteggia fracture [14]. The radial nerve separates into deep and superficial branches in the anterior aspect of the elbow. The deep branch of the radial nerve passes around the radial head and enters the deep and superficial layers of the supinator muscle, then exits the supinator muscle, and travels along the surface of the interosseous membrane toward the distal part of the forearm. When the radial head is dislocated, it can compress the deep branch of the radial nerve, causing nerve damage [15]. In this study, we recorded five patients with radial nerve injury, all of whom had sensory abnormalities in the "tiger's mouth" area and mild limitations in extending their thumbs and wrists. Therefore, when dealing with Monteggia fracture, timely reduction of the radial head to relieve compression can lead to natural recovery of most cases of radial nerve injury, without the need for special treatment.

Different treatment methods can be used for children with Monteggia fracture, primarily determined by the characteristics of the ulnar fracture rather than radial head dislocation [16]. The principle of treatment for acute Monteggia fracture in children is to correct ulnar deformity, maintain ulnar length, and ensure stable reduction of the fracture and humeroradial joint. Bado Type I Monteggia fracture can generally be satisfactorily treated by closed reduction and plaster immobilization [17]. For unstable oblique fractures of the ulna, complete transverse fractures with displacement, or cases where the radial head cannot be reduced or maintained stability after reduction, surgical treatment is a more appropriate choice [5, 18]. In this study, we used percutaneous Kirschner wire or elastic intramedullary nail for fixation according to the location of the ulnar fracture, while the ulnar olecranon and ulnar diaphysis were commonly stabilized with Kirschner wire. The junction of the upper and middle thirds of the ulna is the transition zone between the ulnar shaft and its near-side metaphysis. The medullary canal diameter in this region is significantly widened compared to that of the shaft. ESIN technique cannot achieve the "three-point support" standard for fracture reduction, and increasing the diameter of the elastic nail may not effectively prevent lateral or anterior/posterior displacement of the fracture and may also cause difficulty in distal pin insertion. For such fractures, we pre-bent the elastic nail into an S shape and fixed each end of the S shape to the far and near fragments of the fracture, thereby increasing the contact surface area between the elastic nail and the medullary cavity, achieving a more stable "four-point support," effectively correcting lateral and anterior–posterior displacement of the fracture, and expanding the application range of elastic nail in pediatric Monteggia fracture [9].

Currently, the evaluation of reduction effect in fracture mainly relies on intraoperative fluoroscopy. In a normal elbow joint, the longitudinal axis extension line of the radial neck in a supinated forearm should pass through the center of the humeral head. However, some studies have shown that about 16% of X-ray in children's healthy elbow joint does not meet this criterion [19], and as the elbow joint in children is not yet fully ossified, X-ray cannot directly display the alignment and matching degree of the humeroradial joint [20]. This directly affects the accuracy of this judgment standard. Therefore, more accurate diagnostic methods are needed for the treatment of Monteggia fracture in children. Ultrasound can clearly display the ossification center, epiphyseal end, epiphyseal plate, cortical bone, and articular cartilage of children's bones. It can also accurately judge the alignment of fracture and dislocation [21]. Ultrasound is an optional method for diagnosing distal ulnar and radial fractures in children [22, 23]. Ultrasound guidance can help surgeons perform accurate fracture reduction. The sensitivity and specificity of ultrasound in identifying insufficient reduction of ulnar fractures in children were 100% and 92–93%, respectively [22]. With ultrasound guidance, Bobby achieved a success rate of 94% in the reduction of a closed forearm fracture, which helped to minimize unnecessary damage during the reduction process [24]. Storch et al. [25] reported that ultrasound is the preferred method for assessing the integrity of the articular cartilage hinge in children with undisplaced lateral humeral condyle fractures. Li et al. [26] used ultrasound-guided reduction and Kirschner wire fixation for 44 patients with displaced and rotated lateral humeral condyle fractures, achieving satisfactory reduction and elbow function in 42 patients. Compared with fluoroscopy, it has the enormous advantage of dynamic and multi-angle monitoring, which effectively improves the success rate of closed reduction [8]. Additionally, it has no ionizing radiation, greatly reducing patient radiation exposure during surgery. During surgery, only 1–2 fluoroscopic examinations are necessary for verification of fracture reduction, which greatly shortens the operating time. Additionally, ultrasound machines are small and easy to move and operate, and ultrasound technology allows for more accurate reduction through real-time comparison with the unaffected side's ultrasound image. In our study, ultrasound guidance was used to perform reduction and accurately locate insertion points and angles in 31 children with Monteggia fracture, increasing the success rate of nail placement, avoiding secondary injuries caused by reduction, and obtained satisfactory reduction by observing the reduction from multiple sections. For those who have difficulty maintaining the reduction or those with difficult reduction, ultrasound can be used to check whether there is soft tissue entrapment in the humeroradial joint. If there is entrapment, Kirschner wire can be used to assist in reducing the entrapped soft tissue, and if reduction fails, open reduction can be performed. Radial nerve injury is a common complication of Monteggia fracture. Among the cases in our group, five had concurrent radial nerve injury, which was verified using ultrasound, and none of them underwent open exploration. The symptoms of nerve damage disappeared in these patients after 3 months. For patients with radial nerve rupture, early open exploration can be performed. Consequently, ultrasound is another major advantage in judging the condition of the nerve injury [8].

### Limitations

The sample size included in this report is relatively small, lacking a control group, and the follow-up time is short.

## Conclusion

In conclusion, treating acute Monteggia fracture in children under ultrasound guidance has advantages such as less trauma, less radiation exposure, and better reduction quality and can achieve satisfactory mid-to-long-term clinical efficacy.

## Data Availability

According to reasonable requirements, the corresponding authors will provide original data to support the conclusion of this paper.

## Abbreviation

ESIN: Elastic stable intramedullary nail
